# Key Features of Contemporary Pilot and Feasibility Trials: Protocol for a Methodological Study

**DOI:** 10.1111/aas.70228

**Published:** 2026-03-26

**Authors:** Aske Tøgern, Morten H. Møller, Anders Perner, Maj‐Brit N. Kjær, Ruben J. Eck, Carl T. Anthon, Jehad A. Barakji, Anders Granholm

**Affiliations:** ^1^ Department of Intensive Care Copenhagen University Hospital—Rigshospitalet Copenhagen Denmark; ^2^ Department of Clinical Medicine University of Copenhagen Copenhagen Denmark; ^3^ Department of Internal Medicine University Medical Center Groningen Groningen the Netherlands; ^4^ Section of Biostatistics, Department of Public Health University of Copenhagen Copenhagen Denmark

**Keywords:** feasibility, pilot trial, randomised clinical trial

## Abstract

**Background:**

Randomised clinical trials (RCTs) are costly undertakings requiring careful design, planning and conduct. Failure to achieve timely participant recruitment, separation between intervention groups and adequate follow‐up contributes to waste in research. Pilot RCTs offer researchers the opportunity to examine whether a definitive trial may work as intended, for example, assessing whether the targeted sample size can be recruited, whether protocol adherence and between‐group separation are achievable and whether outcome data can be collected. To maximise their value, more attention to the design, conduct and reporting of pilot RCTs is likely required. Here, we aim to describe and characterise feasibility assessment methodology in a cohort of contemporary pilot RCTs in hospitalised patients.

**Methods:**

In this methodological study, we will describe key features of contemporary pilot RCTs in hospitalised patients across medical and surgical specialties. We will search for pilot/feasibility RCTs published within the last half calendar year, with the aim of including at least 100 RCTs assessing feasibility. If necessary to achieve the minimum sample, the search will be expanded to a maximum of 5 years. We will extract data on key trial characteristics (including area of care and type of intervention and comparator), whether feasibility was assessed and feasibility assessment methodology (feasibility areas assessed, conduct as stand‐alone pilot, use of pre‐defined outcomes and pre‐specified criteria for progression to definitive RCT, adequacy of sample size justification, plans for definitive RCT, assessment of clinical outcomes and whether a definitive trial was deemed feasible). Findings will be summarised using descriptive statistics for all trials and stratified by intervention type, area of care and comparator type.

**Conclusions:**

The outlined methodological study will provide data on key features of contemporary pilot RCTs in hospitalised patients, including the feasibility assessment methodologies employed. This may improve design, conduct and reporting of future pilot RCTs, ultimately increasing their value and reducing research waste.

## 1 Introduction

Randomised clinical trials (RCTs) are the gold‐standard for assessing interventions and thus central in evidence‐based medicine [1, 2]. RCTs are costly and resource‐intensive to design, plan and conduct [3], require substantial time investments by clinicians and researchers, place a burden on participants (e.g., informed consent procedures, trial visits, contacts for follow‐up) and use limited research resources. Fundamental to the success of most RCTs are recruitment of the right participants within a reasonable timeframe, achieving acceptable protocol adherence, obtaining relevant separation between intervention groups in terms of intervention delivery, and acquiring adequate follow‐up data on individual participants.

Even well‐designed RCTs based on rigorous methodology may encounter operational obstacles during conduct [2, 4]. Failure to recruit the planned‐for number of trial participants is widespread [5], which can render RCTs unable to detect intervention effects of the sizes planned for [6]. Further, poor protocol adherence and/or between‐group separation can threaten the internal validity of RCTs [7].

To reduce waste in research, it is important that RCTs that are unlikely to accomplish their aims are identified as early as possible and appropriately modified or abandoned. In clinical research, pilot trials are employed to inform the design and conduct of, and decision to commence with, a definitive larger RCT [8].

Guidance on the conduct and reporting of pilot trials exists [9, 10, 11]. Still, the need for methodologically rigorous pilot trials has been noted [12], and more comprehensive reporting on feasibility parameters has been called for [9, 13]. Previous meta‐research in the field of feasibility studies and pilot trials has focused on specific specialties [14, 15, 16, 17, 18], use in planning RCTs funded by specific funding bodies [19, 20], or specifically addressed use of progression criteria in pilot trials [21, 22, 23].

With the methodological study proposed here, we aim to describe characteristics of and feasibility assessment methodology in a contemporary cohort of pilot RCTs in hospitalised patients.

## 2 Methods

The present protocol is reported according to the *Preferred Reporting Items for Systematic review and Meta‐analysis protocols* (*PRISMA‐P*) statement [24] and the guidelines for reporting meta‐epidemiological methodology research by Murad and Wang [25]. Completed checklists are provided in the Supporting Information (Appendices C and D). At the time of submission of this protocol for publication, the conduct of the methodological study has not commenced.

### 2.1 Eligibility Criteria

We will include randomised ‘pilot’ and/or ‘feasibility’ trials assessing interventions in hospitalised patients (including interventions delivered in the emergency room or prehospital setting if the patient population or clinical condition usually warrant immediate admission to hospital). We will include both completed trials with result publications available, and ongoing trials with only published protocols available. Stand‐alone (i.e., external) and integrated (i.e., internal) pilot trials are equally eligible.

We will exclude trials with cross‐over or cluster randomisation designs, because challenges of conducting such trials are expected to differ in important aspects from those associated with trials using individual, parallel‐group randomisation. We will also exclude trials where the intervention is delivered in a context outside hospital with participants not expected to require immediate hospitalisation (e.g., community‐based interventions), and trials where the intervention is delivered in outpatient clinics. Additionally, we will exclude pilot trials without an available full text protocol or result publication (e.g., only conference abstract or mention in a correspondence/letter is available). We will not exclude trials based on language of publication.

Extended definitions of the eligibility criteria are provided in the Supporting Information (Appendix B).

### 2.2 Information Sources and Search Strategy

It has been recommended to use the term ‘pilot trial’ for the subset of feasibility studies employing a randomisation procedure and testing the processes of the planned RCT in a ‘miniature’ version [9, 11, 12, 26]. In practice, the terms ‘feasibility trial’ and ‘pilot trial’ are often used without clear distinction [9, 11], and for the study proposed here we use the terms interchangeably.

We will systematically search *PubMed* for randomised pilot trials using the terms ‘pilot’ and ‘feasibility’ (restricted to title and abstract) and the Cochrane Highly Sensitive Search Strategy for identifying randomised trials (sensitivity‐maximising version, 2008 revision) [27]. The search strategy has been developed with assistance from the hospital's medical librarian and the full search string is provided in the Supporting Information (Appendix A). We will restrict the search according to date of publication to include pilot RCTs that reflect contemporary approaches.

We will use a quota sampling strategy, where we expect to be able to include a minimum of 100 trials for the full analysis from a search restricted to the last half calendar year (the latter half of 2025) plus the time passed until conduct of the search (e.g., the first months of the year 2026). If less than 100 trials are included through this first search, the search will be expanded consecutively to cover another half year until a minimum of 100 trials have been included for full analysis. If relevant according to the time passed since the initial search, and to preserve the contemporary scope, we may alternatively proceed by expanding the search to cover a period succeeding that covered in the first search.

For each included trial, we will assess protocols and result publications (including publicly available Supporting Information) identified in our search. Where a result publication for an included trial directly references a trial protocol, trial registration or secondary publication reporting on feasibility, we will assess these sources if they are publicly available. We will not search additional repositories (e.g., trial registries) and we will not contact trial authors for additional information.

### 2.3 Study Selection Process

We will screen identified titles/abstracts and full‐text publications regarding potentially eligible trials independently and in duplicate using *Covidence* (Veritas Health Innovation, Melbourne, Australia; www.covidence.org). We will resolve conflicts in the selection process through discussion and, where necessary, by involving a third author.

**FIGURE 1:**
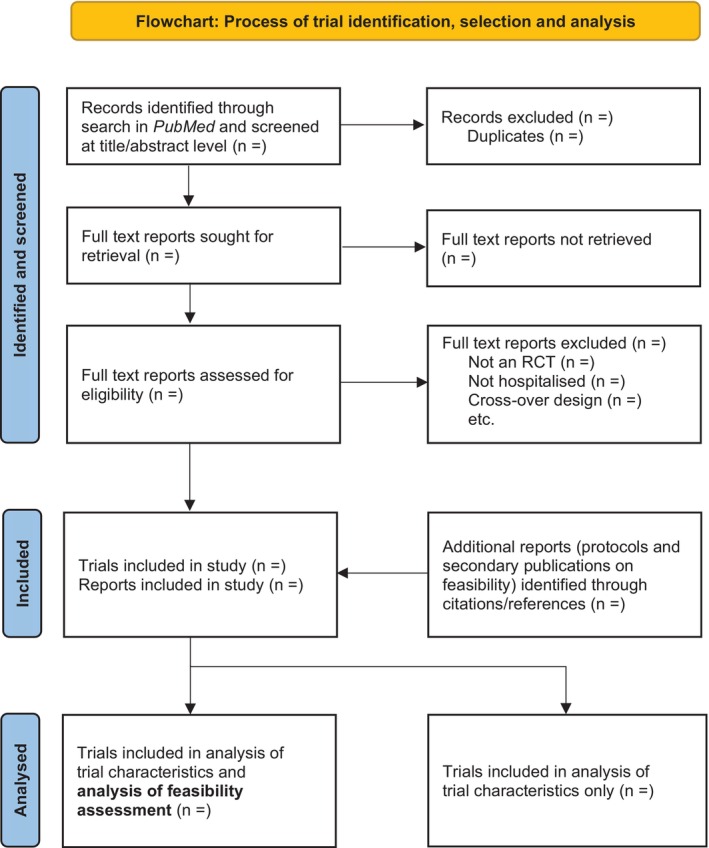
PRISMA flowchart. Flow of trial identification, selection and analysis mock figure. Flowchart adapted from the *Preferred Reporting Items for Systematic Reviews and Meta‐Analyses extension for scoping reviews* (*PRISMA‐ScR*) flowchart [28].

We will present the flow of identified search records and the trial selection (as well as the data extraction process) in a flowchart (Figure 1) adapted from the *Preferred Reporting Items for Systematic Reviews and Meta‐Analyses extension for scoping reviews* (*PRISMA‐ScR*) flowchart [28].

### 2.4 Data Items and Collection

We will extract data independently and in duplicate using the data extraction form provided in the Supporting Information (Appendix E). Disagreements will be solved through discussion and, if needed, with the involvement of a third author. The data extraction form will be piloted on the first 10 trials by at least two authors and revised as required before continuing data extraction.

Data extraction is two‐tiered. For all included RCTs, data on trial characteristics will be extracted (Table 1), including whether feasibility was assessed. For the minimum sample of 100 RCTs where feasibility was assessed, we will also extract data on the feasibility assessment methodology (Table 2). Definitions of all variables and categorisations used during data extraction are provided in the Supporting Information (Appendix B).

**TABLE 1:** Trial characteristics (mock table).

Variable	All RCTs (*N* = #)	*Stratified by feasibility assessment*		*Stratified by intervention type*		
		RCTs not assessing feasibility (*N* = #)	RCTs assessing feasibility (N = #)	Drug (among RCTs assessing feasibility) (*N* = #)	Device (among RCTs assessing feasibility) (*N* = #)	Management (among RCTs assessing feasibility) (*N* = #)
Year of publication^a^						
2025	*n* (#.#%)	*n* (#.#%)	*n* (#.#%)	*n* (#.#%)	*n* (#.#%)	*n* (#.#%)
Number of sites	## (## to ##) [## to ##]	## (## to ##) [## to ##]	## (## to ##) [## to ##]	## (## to ##) [## to ##]	## (## to ##) [## to ##]	## (## to ##) [## to ##]
Number of participants	## (## to ##) [## to ##]	## (## to ##) [## to ##]	## (## to ##) [## to ##]	## (## to ##) [## to ##]	## (## to ##) [## to ##]	## (## to ##) [## to ##]
Area of care^b^						
Critical care	*n* (#.#%)	*n* (#.#%)	*n* (#.#%)	*n* (#.#%)	*n* (#.#%)	*n* (#.#%)
Emergency including prehospital care	*n* (#.#%)	*n* (#.#%)	*n* (#.#%)	*n* (#.#%)	*n* (#.#%)	*n* (#.#%)
Anaesthesia and perioperative care including surgical interventions	*n* (#.#%)	*n* (#.#%)	*n* (#.#%)	*n* (#.#%)	*n* (#.#%)	*n* (#.#%)
Non‐critical non‐emergent interventions on the ward	*n* (#.#%)	*n* (#.#%)	*n* (#.#%)	*n* (#.#%)	*n* (#.#%)	*n* (#.#%)
Other areas	*n* (#.#%)	*n* (#.#%)	*n* (#.#%)	*n* (#.#%)	*n* (#.#%)	*n* (#.#%)
Participant age group						
Adult	*n* (#.#%)	*n* (#.#%)	*n* (#.#%)	*n* (#.#%)	*n* (#.#%)	*n* (#.#%)
Children	*n* (#.#%)	n (#.#%)	*n* (#.#%)	*n* (#.#%)	*n* (#.#%)	*n* (#.#%)
Mixed	n (#.#%)	*n* (#.#%)	*n* (#.#%)	*n* (#.#%)	*n* (#.#%)	*n* (#.#%)
Number of intervention groups^c^						
2	*n* (#.#%)	*n* (#.#%)	*n* (#.#%)	*n* (#.#%)	*n* (#.#%)	*n* (#.#%)
More than 2	*n* (#.#%)	*n* (#.#%)	*n* (#.#%)	*n* (#.#%)	*n* (#.#%)	*n* (#.#%)
Intervention type						
Drug	*n* (#.#%)	*n* (#.#%)	*n* (#.#%)	*n* (100.0%)	0 (0.0%)	0 (0.0%)
Device	*n* (#.#%)	*n* (#.#%)	*n* (#.#%)	0 (0.0%)	*n* (100.0%)	0 (0.0%)
Management	*n* (#.#%)	*n* (#.#%)	*n* (#.#%)	0 (0.0%)	0 (0.0%)	n (100.0%)
Comparator type						
Active comparator	*n* (#.#%)	*n* (#.#%)	*n* (#.#%)	*n* (#.#%)	*n* (#.#%)	*n* (#.#%)
No treatment	*n* (#.#%)	*n* (#.#%)	*n* (#.#%)	*n* (#.#%)	*n* (#.#%)	*n* (#.#%)
Placebo or sham intervention	*n* (#.#%)	*n* (#.#%)	*n* (#.#%)	*n* (#.#%)	*n* (#.#%)	*n* (#.#%)
Blinding						
Participants	*n* (#.#%)	*n* (#.#%)	*n* (#.#%)	*n* (#.#%)	*n* (#.#%)	*n* (#.#%)
Care providers	*n* (#.#%)	*n* (#.#%)	*n* (#.#%)	*n* (#.#%)	*n* (#.#%)	*n* (#.#%)
Outcome assessors	*n* (#.#%)	*n* (#.#%)	*n* (#.#%)	*n* (#.#%)	*n* (#.#%)	*n* (#.#%)
Full protocol published	*n* (#.#%)	*n* (#.#%)	*n* (#.#%)	*n* (#.#%)	*n* (#.#%)	*n* (#.#%)
Pilot results published	*n* (#.#%)	*n* (#.#%)	*n* (#.#%)	*n* (#.#%)	*n* (#.#%)	*n* (#.#%)
Feasibility assessed	*n* (#.#%)	0 (0.0%)	*n* (100.0%)	*n* (100.0%)	*n* (100.0%)	*n* (100.0%)

*Note:* Trial characteristics—total for all RCTs, and stratified by feasibility assessment and, for RCTs assessing feasibility, intervention type.

Categorical and binary variables are presented as counts (percentages). Numerical variables are presented as medians (interquartile range) [full ranges].

Trial characteristics to be extracted are DOIs*, trial name*, year of publication, country of origin*, number of sites, start of participant recruitment*, end of participant recruitment*, number of participants randomised, area of care, participant age group, number of intervention groups, intervention type, comparator type, blinding, publication of full protocol, publication of pilot trial results and whether feasibility was assessed (i.e., assessment of trial design/conduct/process variables and not (only) of clinical outcomes registered at the individual participant level). Characteristics marked with an asterix (*) are not shown in the table and will be reported in an appendix to the result manuscript. Definitions of all variables and categorisations are provided in the Supporting Information (Appendix B).

Abbreviations: DOI, digital object identifiers; RCT, randomised clinical trial.

^a^ Year of publication is registered for each RCT and will be reported in an appendix to the result manuscript. Because most RCTs are expected to be published in 2025, we will present it as a categorical variable with two levels (publication in 2025 or not). If a substantial number of RCTs are not published in 2025, we may choose to present it differently.

^b^ The categorisation of care area is subject to change during data extraction if deemed relevant, that is, new categories may be defined if a lot of trials are within areas of care not meaningfully covered by the present categories. Area of care for each included RCT will be presented in detail in a separate table in an appendix to the result manuscript.

^c^ Number of intervention groups is presented as a categorical variable with two levels (two or more than two). The raw number of intervention groups in each RCT will be registered and reported in an appendix to the result manuscript, and if deemed relevant we will report counts and percentages for more than two levels.

**TABLE 2:** Feasibility assessment methodology (mock table).

Variable	RCTs assessing feasibility (*N* = #)	Stratified by intervention type		
		Drug (*N* = #)	Device (*N* = #)	Management (N = #)
Stand‐alone pilot	*n* (#.#%)	*n* (#.#%)	*n* (#.#%)	*n* (#.#%)
Feasibility area(s) assessed^a^				
Recruitment	*n* (#.#%)	*n* (#.#%)	*n* (#.#%)	*n* (#.#%)
Randomisation procedure	*n* (#.#%)	*n* (#.#%)	*n* (#.#%)	*n* (#.#%)
Consent	*n* (#.#%)	*n* (#.#%)	*n* (#.#%)	*n* (#.#%)
Blinding procedures	*n* (#.#%)	*n* (#.#%)	*n* (#.#%)	*n* (#.#%)
Protocol adherence	*n* (#.#%)	*n* (#.#%)	*n* (#.#%)	*n* (#.#%)
Between‐group separation	*n* (#.#%)	*n* (#.#%)	*n* (#.#%)	*n* (#.#%)
Retention and attrition	*n* (#.#%)	*n* (#.#%)	*n* (#.#%)	n (#.#%)
Other	*n* (#.#%)	*n* (#.#%)	*n* (#.#%)	*n* (#.#%)
Outcomes pre‐defined	*n* (#.#%)	*n* (#.#%)	*n* (#.#%)	*n* (#.#%)
Progression criteria pre‐specified	*n* (#.#%)	*n* (#.#%)	*n* (#.#%)	*n* (#.#%)
Sample size adequately justified	*n* (#.#%)	*n* (#.#%)	*n* (#.#%)	*n* (#.#%)
Preparing for larger definitive RCT	*n* (#.#%)	*n* (#.#%)	*n* (#.#%)	*n* (#.#%)
Clinical outcomes assessed				
All intervention groups combined	*n* (#.#%)^b^	*n* (#.#%)^b^	*n* (#.#%)^b^	*n* (#.#%)^b^
Separately by intervention group	*n* (#.#%)^b^	*n* (#.#%)^b^	*n* (#.#%)^b^	*n* (#.#%)^b^
No	*n* (#.#%)^b^	*n* (#.#%)^b^	*n* (#.#%)^b^	*n* (#.#%)^b^
Trial deemed feasible				
Yes	*n* (#.#%)^b^	*n* (#.#%)^b^	*n* (#.#%)^b^	*n* (#.#%)^b^
With modifications	*n* (#.#%)^b^	*n* (#.#%)^b^	*n* (#.#%)^b^	*n* (#.#%)^b^
No	*n* (#.#%)^b^	*n* (#.#%)^b^	*n* (#.#%)^b^	n (#.#%)^b^

*Note:* Feasibility assessment methodology—total for RCTs assessing feasibility and stratified by intervention type.

Categorical and binary variables are presented as counts (percentages).

Abbreviation: RCT, randomised clinical trial.

^a^ The categorisation of feasibility areas is subject to change during data extraction if deemed relevant, that is, new categories may be defined if a lot of trials assess areas not meaningfully covered by the present categories. Area of feasibility assessment for each included RCT will be presented in separate table in an appendix to the result manuscript.

^b^ Percentages to be reported are the percentages calculated using the number of *trials with published pilot trial results* as the denominator.

### 2.5 Data Synthesis

Data on trial characteristics will be summarised using descriptive statistics, that is, counts and percentages for categorical variables, and medians with interquartile and full ranges for numerical variables. We will present these across all included pilot RCTs as well as separately for trials assessing feasibility and, among those assessing feasibility, stratified by intervention type (drug, device or management), area of care (critical care, emergency including prehospital care, anaesthesia and perioperative care including surgical interventions, non‐critical non‐emergent interventions on the ward, other areas) and comparator type (active comparator, no treatment, placebo or sham intervention) (Table 1 and Tables S1 and S2 in Appendix F in the Supporting Information).

Data on feasibility assessment methodologies will be summarised using descriptive statistics (as above) (Table 2). For the variables in this section, we will use the number of included RCTs assessing feasibility (i.e., the number of RCTs included for the full analysis) as denominator. Two variables in this section (whether clinical outcomes were assessed, and whether investigators deemed the trial as feasible) are applicable only to pilot RCTs with published results, and for these the number of included trials with published results will be used as the denominator. Results stratified by intervention type, area of care and comparator type will also be presented (Table 2 and Tables S3 and S4 in Appendix F in the Supporting Information).

### 2.6 Ethics

This methodological study does not require ethical approval as no health‐related or other individual‐participant outcomes are assessed, and as all data included are already in the public domain.

### 2.7 Reporting and Dissemination

Work to develop guidelines for reporting of methodological studies is ongoing [29]. Currently, a guideline for reporting meta‐epidemiological studies has been adapted from the PRISMA checklist [25]. Although the study proposed here is not meta‐epidemiological, we have developed the protocol and will report results according to this guideline, as we believe it currently represents the best option for clear and transparent reporting of methodology research. Where reporting of results deviates from the protocol, we will label this clearly and provide rationales.

We aim to disseminate the results from this study through publication in an international peer‐reviewed scientific journal and presentation at conferences.

## 3 Discussion

For pilot RCTs to realise their potential for reducing waste in research, they should be designed and conducted with methodological rigour and reported comprehensively and transparently. The methodological study proposed in this protocol will describe the characteristics of a contemporary cohort of pilot RCTs in hospitalised patients and the employed feasibility assessment methodologies. Our results may help trialists improve the design, conduct, and reporting of future pilot RCTs and, ultimately, increase pilot RCTs' value to the research community, patients and society.

### 3.1 Strengths and Limitations

The outlined methodological study has several strengths. To promote transparency, reproducibility and future synthesis of the knowledge base in the field, we will conduct the study according to aims and methods described in this protocol and aligning with available guidance for reporting methodological research [25]. Feasibility research is an emerging field with still relatively recently published guidelines for reporting [11], increasing numbers of publications designated with the terms ‘pilot’ and ‘feasibility’ and growing interest in and recognition of the scientific merit of feasibility work [10]. To increase the potential value to trialists in several areas of medicine, we will focus on methodologies employed in a broad contemporary cohort of pilot RCTs. Our stratification of results by key trial characteristics might elucidate patterns and point to areas where increased attention in the design and reporting of pilot RCTs is needed.

Our proposed study is not without limitations. Authors may sometimes apply the term ‘pilot’ to their RCT only after it has failed to provide conclusive answers to the clinical questions motivating its conduct, even if that term was never used in the trial protocol. Pilot RCTs may never reach publication [30], perhaps particularly those where investigators deem a subsequent trial unfeasible. Due to inconsistent terminology [9] and imperfect indexing, we may miss trials that could be identified as pilot RCTs. With appearance of journals dedicated to feasibility work [30] and increasing recognition of its value, we believe our search will cover the majority of published pilot RCTs. Feasibility work is increasingly conducted as integrated pilot phases [10] where feasibility is not always reported independently of the main trial results, but missing reports on such work may represent less of a problem to clinical trialists as this approach is usually chosen when the degree of uncertainty is deemed lower [10].

## 4 Conclusions

The methodological study outlined in this protocol will provide an overview of a contemporary cohort of pilot RCTs and the employed feasibility assessment methodologies, which can inform the design, planning and conduct of future trials.

## Author Contributions

**Anders Granholm**, **Anders Perner**, **Aske Tøgern**, **Maj‐Brit N. Kjær**, **Morten H. Møller**, and **Ruben J. Eck:** conceptualization. **Anders Granholm**, **Anders Perner**, **Maj‐Brit N. Kjær**, **Morten H. Møller**, and **Ruben J. Eck:** supervision. **Aske Tøgern:** writing – original draft and project administration. All authors: writing – review and editing and methodology.

## Funding

Aske Tøgern's salary is paid by a grant from the Novo Nordisk Foundation. All authors are involved with the INCEPT research programme, which has received funding from the Novo Nordisk Foundation (NNF23OC0085106) and Sygeforsikringen ‘danmark’ (2020‐0320), with additional support by Grosserer Jakob Ehrenreich og Hustru Grete Ehrenreichs Fond, Dagmar Marshalls Fond and Savværksejer Jeppe Juhl og hustru Ovita Juhls Mindelegat, and with INCEPT‐Albumin and INCEPT‐Thromboprophylaxis domains, which have received funding from Danmarks Frie Forskningsfond (4308‐00210B) and the Netherlands Thrombosis Foundation, respectively. None of the funders have had any influence on the design of the study or the development of the protocol, and none will have any influence on the conduct, analysis or reporting of the study. None of the funders will have ownership of any data.

## Conflicts of Interest

All authors are involved with ongoing randomised clinical trials assessing feasibility, including INCEPT (www.incept.dk) and its INCEPT‐Albumin and INCEPT‐Thromboprophylaxis domains, which encompass integrated feasibility phases.

## Supporting information


**Appendix A:** Search strategy.
**Appendix B:** Eligibility and variable definitions.
**Appendix C:** PRISMA‐P Checklist.
**Appendix D:** Checklist for reporting of meta‐epidemiological studies.
**Appendix E:** Data extraction form.
**Appendix F:** Supplemental tables.

## Data Availability

Data sharing not applicable to this article as no datasets were generated or analysed during the current study.
