# Supplementary material for: Key Features of Contemporary Pilot and Feasibility Trials: Protocol for a Methodological Study
Source: Acta Anaesthesiol Scand. 2026 Mar 26;70(5):e70228. doi: 10.1111/aas.70228 (PMC13021571; doi:10.1111/aas.70228)
Supplement: Supplementary file 1 — Appendix A: Search strategy. Appendix B: Eligibility and variable definitions. Appendix C: PRISMA‐P Checklist. Appendix D: Checklist for reporting of meta‐epidemiological studies. Appendix E: Data extraction form. Appendix F: Supplemental tables. [file AAS-70-0-s001.pdf]

SUPPORTING INFORMATION

SUPPLEMENT TO:

Keys features of contemporary pilot and feasibility trials: protocol for a methodological study

Aske Tøgern[1], Morten H. Møller[1,2], Anders Perner[1,2], Maj-Brit N. Kjær[1], Ruben J. Eck[3], Carl T. Anthon[1], Jehad A. Barakji[1], Anders Granholm[1,4]

[1] Department of Intensive Care, Copenhagen University Hospital - Rigshospitalet, Copenhagen, Denmark

[2] Department of Clinical Medicine, University of Copenhagen, Copenhagen, Denmark

[3] Department of Internal Medicine, University Medical Center Groningen, Groningen, the Netherlands

[4] Section of Biostatistics, Department of Public Health, University of Copenhagen, Copenhagen, Denmark

Corresponding author

Aske Tøgern, MD, PhD-student

Department of Intensive Care 4131, Copenhagen University Hospital - Rigshospitalet, Blegdamsvej 9, 2100 Copenhagen, Denmark

Email: aske.toegern@regionh.dk

Contents

Appendix A: Search strategy 3
Appendix B: Eligibility and variable definitions 4
Appendix C: PRISMA-P Checklist 9
Appendix D: Checklist for reporting of meta-epidemiological studies 11
Appendix E: Data extraction form 16
Appendix F: Supplemental tables 17
References 23

Appendix A: Search strategy

PubMed will be searched using the Cochrane Highly Sensitive Search Strategy for identifying randomised trials (sensitivity-maximising version, 2008 revision).[1]

The following search string will be used, except that the dates of publication will be changed to match a coverage of the last ful calendar year (the year 2025) plus the time passed until conduct of the search:

(((randomized controlled trial[pt] OR controlled clinical trial[pt] OR randomized[tiab] OR placebo[tiab] OR drug therapy[sh] OR randomly[tiab] OR trial[tiab] OR groups[tiab] NOT (animals [mh] NOT humans [mh])))

AND

((pilot[Title/Abstract] OR feasibility[Title/Abstract])))

AND

(("2025/01/01"[Date - Publication] : "2025/12/31"[Date - Publication]))

Appendix B: Eligibility and variable definitions

This list contains definitions of the criteria used for assessing eligibility during screening of trials, and of the variables used in extraction of both trial characteristics data and feasibility assessment methodology data.

Eligibility

The population of studies that are of interest for this methodological study are *randomised pilot trials concerned with interventions in hospitalised patients*.

Randomised pilot trials: any trials labelled with the term(s) “pilot” and/or “feasibility” in the title and/or abstract of the protocol and/or result publication, and where participants are individually and parallelly randomised (i.e., cluster randomisation or cross-over designs do not fulfill the criterion). If the pilot or feasibility term applies to an integrated phase of a larger randomised clinical trial (RCT), this internal pilot phase will be assessed similarly to how we will assess stand-alone (external) pilot trials. We will consider for inclusion both completed trials with available result publications, and ongoing trials with only protocols published.

Interventions in hospitalised patients: any intervention in patients (e.g., therapeutic, diagnostic or prognostic) that is usually delivered during hospital stay (as deemed by the authors of this methodological study). Interventions that are started in a prehospital setting or during patient evaluation in an emergency room, but that are continued during subsequent hospital admission, will be considered as fulfilling this criterion. Interventions delivered solely in the emergency room or prehospital setting, but where immediate admission to hospital is usually warranted due to characteristics of the patient population and/or clinical condition (as deemed by the authors of this methodological study), will also be considered as fulfilling this criterion. Both pharmacological (drugs and fluids) and non-pharmacological interventions (devices and management) will be considered.

Trial characteristics

DOI(s): digital object identifier(s) for protocol publications and/or result publications associated with the pilot RCT (for internal pilot phases, the DOI(s) refer(s) to the publication(s) describing the pilot phase).

Trial name: name (or acronym) of trial; where no name is used for the trial, we will instead register first author of principal publication (result publication if this is available, otherwise protocol publication).

Year of publication: calendar year of principal publication (result publication if this is available, otherwise protocol publication); we will register year of publication (not date of indexing in *PubMed*) and we will use date of the first publication if more than one date of publication is provided (i.e., electronic and printed publications).

Country of origin: country where the pilot RCT was/is conducted (for international trials, we will instead register location of the sponsor).

Number of sites: number of sites enrolling participants for the pilot RCT (where no result publication is available, we will instead register the number of sites expected to enroll participants for the pilot RCT, if this is available).

Start of participant recruitment: the calendar year and month, where recruitment of participants for the pilot RCT started (where no result publication is available, we will instead register the expected start of participant recruitment, if this is available).

End of participant recruitment: the calendar year and month, where recruitment of participants for the pilot RCT ended (where no result publication is available, we will instead register the expected end of participant recruitment, if this is available).

Number of participants: number of randomised participants in the pilot RCT (across all intervention groups) (where no result publication is available, we will instead register the targeted sample size for the pilot RCT).

Area of care: the clinical specialty/environment in which the intervention is delivered categorised as
- critical care (i.e., interventions delivered in an intensive/critical care unit),
- emergency including prehospital care (i.e., interventions delivered in an emergent setting outside the intensive/critical care unit, e.g., prehospital, emergency room, emergency surgery or emergent setting inside a hospital),
- anaesthesia and perioperative care including surgical interventions (i.e., interventions delivered in the operating room or perioperative/postanaesthetic unit but in a non-emergent setting),
- non-critical non-emergent interventions on the ward (e.g., medical treatment or physical therapy on the ward),
- other areas (e.g., diagnostic/interventional procedures in the radiology suite).

The categories above are considered mutually exclusive. An intervention that is surgical or related to anaesthesia but delivered in an emergent setting will be registered as “emergency including pre-hospital care”, because the condition of emergency is believed to confer important implications for design and conduct of RCTs.

Categories of care area are subject to change during data extraction if deemed relevant, i.e., if a lot of trials are within areas of care not meaningfully covered in the present categories, new categories may be defined. For each included trial, details of the area of care will be registered (as unstructured text data) and presented in an appendix to the result manuscript.

Participant age group: enrolled patient population described as adults, children or mixed (including both children and adults); we will register this according to the definition used by the authors of the pilot RCT (if the authors provide no definition of the trial population, we will register based on the available patient characteristics).

Number of intervention groups: total number of intervention groups in the pilot RCT; we will register and present in an appendix to the result manuscript the raw number of groups, but as we expect the majority to be 2-group trials, we plan to present data in the manuscript as a categorical variable with two levels (2 or more than 2 groups); if many trials have three or more groups, we will present data in the manuscript as a categorical variable with more than two levels.

Intervention type: intervention of interest categorised as either drug (including fluids), device (e.g., equipment for treating or caring for patients), or management (including both surgery and other invasive procedures, as well as clinical management strategies, e.g., protocols for treatment or care bundles for specific conditions). The categories are considered mutually exclusive.

Comparator type: the intervention of interest is compared to an active comparator (including standard of care or routine care), no treatment, or a placebo or sham intervention. The categories are considered mutually exclusive. A trial with a placebo or sham intervention group will be registered as "placebo or sham intervention", even if another group is no treatment or an active comparator. A trial with a no treatment group will be registered as "no treatment", even if another group is an active comparator.

Blinding: participants blinded to intervention allocation (yes/no), care providers blinded to intervention allocation (yes/no), and outcome assessors blinded to intervention allocation (yes/no) (the outcome here being any clinical outcome registered at the individual participant level); for blinding of outcome assessors, we will register as "yes" if any clinical outcome is assessed by a blinded assessor (i.e., if more than one clinical outcome is registered and assessment of at least one of them is blinded, we will register blinding of outcome assessor as "yes").

Publication of full protocol: full protocol for the pilot RCT published in scientific journal (yes/no); full protocols that are available only as a supplement to a result publication will be registered as "yes"; only protocols that are either identified directly by our search strategy or referenced in a pilot RCT result publication identified by our search (i.e., either referenced to as a separate publication or as a full protocol available on a website) will be considered; for integrated pilot phases of larger RCTs

(internal pilots), this item will be registered as “yes” if a published protocol for the larger main RCT is available. Trial registrations will not be considered full protocols, unless a separate full protocol is digitally accessible from within the trial registration system.

Publication of pilot trial results: results of the pilot RCT have been published in scientific journal (yes/no).

Assessment of feasibility: protocol publication and/or result publication contain any assessment of trial design/conduct/process parameters (yes/no) (this item covers any such evaluation mentioned in the papers); mention only in the title and/or abstract is not sufficient to be registered as “yes”; assessment of clinical efficacy/comparative effectiveness and/or safety/tolerability will not be considered assessments of feasibility.

Patient population: patient population examined in the RCT (e.g., specific clinical conditions) (registered as unstructured text data).

Interventions: clinical intervention and any active comparator examined in the RCT (registered as unstructured text data).

Feasibility assessment methodology

Stand-alone pilot RCT: the feasibility assessment is conducted as a stand-alone (i.e., external) pilot trial (yes/no); stand-alone pilot RCTs are contrasted with an integrated (i.e., internal) pilot phase of an RCT.

Feasibility area(s) assessed: the feasibility assessment(s) relate to recruitment (yes/no), randomisation procedure (yes/no), consent (yes/no), blinding procedures (yes/no), protocol adherence (yes/no), between-group separation (yes/no), retention and attrition (yes/no), other (yes/no). The categories are not mutually exclusive, and an area of feasibility assessment can be registered as related to e.g., both consent and recruitment. Categories of assessed feasibility area are subject to change during data extraction if deemed relevant, i.e., if a lot of trials assess areas not meaningfully covered in the present categories, new categories may be defined. For each included trial, details of the assessed feasibility area will be registered (as unstructured text data) and presented in an appendix to the result manuscript.

Feasibility outcomes pre-defined: the assessment of feasibility involves specific outcomes that are clear and pre-defined (either in trial registration and/or published protocol, or similar pre-definition of feasibility outcomes is described in the result publication) (yes/no).

Feasibility assessment using pre-specified progression criteria: feasibility is evaluated according to criteria for progression to a larger definitive RCT that are pre-specified (in trial registration and/or published protocol, or similar pre-specification of progression criteria is described in the result publication) (yes/no).

Adequate justification of the pilot RCT (or pilot phase) sample size: the sample size in the pilot RCT (or pilot phase) is provided with consideration of assessing feasibility (yes/no); for ongoing trials without published results, we will assess the justification provided for the targeted sample size; justifying the sample size with consideration of clinical effect size estimates will not be deemed adequate.

Pilot RCT conducted in preparation for a larger definitive RCT: protocol publication and/or result publication reference plans for a larger definitive RCT (yes/no) (this item covers any mentions of such in the papers); for integrated pilot phases in a larger RCT (i.e., internal pilots), we will by default consider the value to be “yes”.

Assessment of clinical outcomes: the pilot RCT result publication includes assessment of clinical outcomes registered at the individual participant level, i.e., efficacy/effectiveness and/or safety/tolerability outcomes (across all intervention groups in the RCT combined, separately by each intervention group in the RCT, or no assessment of clinical outcomes). The categories are mutually exclusive, and a trial assessing and reporting clinical outcomes both aggregated across all intervention groups and separately by intervention group will be registered as “separately by intervention group”.

Trial deemed by investigators as feasible: the pilot RCT investigators summarise/conclude that results of the feasibility assessment(s) indicate overall feasibility of proceding with a larger definitive RCT (yes/with modifications/no).

Appendix C: PRISMA-P Checklist

Completed *PRISMA-P* 2015 Checklist [2] as applicable for this protocol (with numbers in red indicating page number on which the item is reported) (NA = not applicable).

PRISMA-P (Preferred Reporting Items for Systematic review and Meta-Analysis Protocols) 2015 checklist: recommended items to address in a systematic review protocol*

Section and topic	Item No	Checklist item
**ADMINISTRATIVE INFORMATION**		
Title:		
Identification	1a	Identify the report as a protocol of a systematic review - NA
Update	1b	If the protocol is for an update of a previous systematic review, identify as such - NA
Registration	2	If registered, provide the name of the registry (such as PROSPERO) and registration number - NA
Authors:		
Contact	3a	Provide name, institutional affiliation, e-mail address of all protocol authors; provide physical mailing address of corresponding author - 1
Contributions	3b	Describe contributions of protocol authors and identify the guarantor of the review - 9
Amendments	4	If the protocol represents an amendment of a previously completed or published protocol, identify as such and list changes; otherwise, state plan for documenting important protocol amendments - NA
Support:		
Sources	5a	Indicate sources of financial or other support for the review - 9
Sponsor	5b	Provide name for the review funder and/or sponsor - NA
Role of sponsor or funder	5c	Describe roles of funder(s), sponsor(s), and/or institution(s), if any, in developing the protocol - 9
**INTRODUCTION**		
Rationale	6	Describe the rationale for the review in the context of what is already known - 3
Objectives	7	Provide an explicit statement of the question(s) the review will address with reference to participants, interventions, comparators, and outcomes (PICO) - 3
**METHODS**		

Eligibility criteria	8	Specify the study characteristics (such as PICO, study design, setting, time frame) and report characteristics (such as years considered, language, publication status) to be used as criteria for eligibility for the review – 4, Supporting Information
Information sources	9	Describe all intended information sources (such as electronic databases, contact with study authors, trial registers or other grey literature sources) with planned dates of coverage - 4-5, Supporting Information
Search strategy	10	Present draft of search strategy to be used for at least one electronic database, including planned limits, such that it could be repeated – 4-5, Supporting Information
Study records:		
Data management	11a	Describe the mechanism(s) that will be used to manage records and data throughout the review - 5
Selection process	11b	State the process that will be used for selecting studies (such as two independent reviewers) through each phase of the review (that is, screening, eligibility and inclusion in meta-analysis) - 5
Data collection process	11c	Describe planned method of extracting data from reports (such as piloting forms, done independently, in duplicate), any processes for obtaining and confirming data from investigators – 5, Supporting Information
Data items	12	List and define all variables for which data will be sought (such as PICO items, funding sources), any pre-planned data assumptions and simplifications – 5, Supporting Information
Outcomes and prioritization	13	List and define all outcomes for which data will be sought, including prioritization of main and additional outcomes, with rationale – 5, Supporting Information
Risk of bias in individual studies	14	Describe anticipated methods for assessing risk of bias of individual studies, including whether this will be done at the outcome or study level, or both; state how this information will be used in data synthesis - NA
Data synthesis	15a	Describe criteria under which study data will be quantitatively synthesised – 6
	15b	If data are appropriate for quantitative synthesis, describe planned summary measures, methods of handling data and methods of combining data from studies, including any planned exploration of consistency (such as I^2, Kendall's τ) – 6
	15c	Describe any proposed additional analyses (such as sensitivity or subgroup analyses, meta-regression) – 6
	15d	If quantitative synthesis is not appropriate, describe the type of summary planned - NA
Meta-bias(es)	16	Specify any planned assessment of meta-bias(es) (such as publication bias across studies, selective reporting within studies) - NA
Confidence in cumulative evidence	17	Describe how the strength of the body of evidence will be assessed (such as GRADE) - NA

*** It is strongly recommended that this checklist be read in conjunction with the PRISMA-P Explanation and Elaboration (cite when available) for important clarification on the items. Amendments to a review protocol should be tracked and dated.** **The copyright for PRISMA-P (including checklist) is held by the PRISMA-P Group and is distributed under a Creative Commons Attribution Licence 4.0.**

Appendix D: Checklist for reporting of meta-epidemiological studies

Completed checklist for reporting methodology research[3] as it applies to this protocol (NA = not applicable).

Table 1

Proposed items to be used for reporting methodology research, adapted from the PRISMA Checklist (http://prisma-statement.org/PRISMAStatement/Checklist.aspx)

Section/topic	Proposed item to be used in methodology research	Reported on page #
**Title**		
Title	Identify the report as a meta-epidemiologic study.	1
**Abstract**		
Structured summary	Provide a structured summary that includes the background of the topic, goal of the study, data sources, method of data selection, appraisal and synthesis methods, results, limitations, conclusions and implications of key findings.	2
**Introduction**		
Rationale	Describe the rationale for the meta-epidemiological study in the context of what is already known.	3

Section/topic	Proposed item to be used in methodology research	Reported on page #
Objectives	Provide an explicit statement of the goal of the meta-epidemiological study and the hypothesis being empirically tested.	3
**Methods**		
Protocol	Indicate if a protocol exists, if and where it can be accessed (eg, Web address). Registration of a protocol is not mandatory.	NA
Eligibility criteria	Specify study characteristics used as criteria for eligibility with a rationale.	4, Supporting Information
Information sources	Describe all information sources (eg, databases with dates of coverage, contact with experts to identify additional studies, Internet searches) and search date.	4-5
Search	Present full electronic search strategy for at least one database, including any limits used, such that it could be repeated. Search is commonly not driven by a clinical question.	4-5, Supporting Information
Study selection	Describe the process for selecting studies for inclusion (ie, how many reviewers selected studies, reviewing in duplicate or by single individuals).	5, 12

Section/topic	Proposed item to be used in methodology research	Reported on page #
Data collection process	Describe method of data extraction from reports (eg, piloted forms, independently, in duplicate) and any processes used for manipulating data or obtaining and confirming data from investigators.	5, Supporting Information
Data items	List and define all variables for which data were sought and any assumptions and imputations made.	5, Supporting Information
Risk of bias in individual studies	If risk of bias assessment of individual studies was relevant to the analysis, describe the items used and how this information is to be used during data synthesis.	NA
Summary measures	State the principal summary measures (eg, ratio of risk ratios, difference in means) and explain its meaning and direction to readers.	6
Synthesis of results	Describe the statistical or descriptive methods of synthesis including measures of consistency if relevant. If applicable, describe the development of statistical or simulation modelling based on theoretical background. Describe and justify assumptions and computational approximations. Describe methods of additional analyses (eg, sensitivity or subgroup analyses, meta-regression), if done, indicating which were prespecified.	6

Section/topic	Proposed item to be used in methodology research	Reported on page #
**Results**		
Study selection	Give numbers of studies assessed for eligibility and included in the study, with reasons for exclusions at each stage, ideally with a flow diagram. Present a measure of inter-reviewer agreement (eg, kappa statistic).	12
Study characteristics	For each study, present characteristics for which data were extracted and provide the citations. Clinical characteristics may not always be relevant.	NA
Risk of bias within studies	If risk of bias assessment of individual studies was used in the meta-epidemiological analysis, report risk of bias indicators of each study to allow replication of findings.	NA
Results of individual studies	Present data elements used in the meta-epidemiological analysis from each study (results of clinical outcomes may not be relevant).	NA
Synthesis of results	Present results of statistical analysis done, including measures of precision and measures of consistency. Present validity of assumptions and fit of statistical or simulation modelling, if applicable.	13-15
Additional analysis	Give results of additional analyses, if done (eg, sensitivity or subgroup analyses, meta-regression).	13-15, Supporting Information

Section/topic	Proposed item to be used in methodology research	Reported on page #
**Discussion**		
Summary of evidence	Summarise the main findings and compare them with existing knowledge about the topic. The quality of evidence may not be relevant; however, investigators should describe their certainty in the results to readers.	7
Limitations	Discuss limitations at research methodology level (eg, likelihood of reporting or publication bias).	7
Conclusions	Provide general interpretation of the results and implications for future research. Provide any plausible impact on clinical practice.	8
**Funding**		
Funding	Describe sources of funding for the methodology research and role of funders.	9

Appendix E: Data extraction form

Variables and the associated data types for the data extraction.

Variable	Trial_ID	DOI(s)	Name	Publication_year	Country	Sites	Recruitment_start	Recruitment_end
Data type	Integer	Text	Text	Date	Text	Integer	Date	Date

Participants	Area_care	Age_group	Intervention_groups	Intervention_type	Comparator_type	Blinding
Integer	Categorical	Categorical	Integer	Categorical	Categorical	Composite, logical

Protocol	Results	Feasibility	Population_patient	Intervention
Logical	Logical	Logical	Text	Text

Standalone	Areas_feasibility	Outcomes	Criteria_progression	Sample_size_justification
Logical	Categorical	Logical	Logical	Logical

Definitive_trial	Clinical_outcomes_assessed	Feasible	Notes
Logical	Categorical	Categorical	Text

Appendix F: Supplemental tables

Supplemental tables (mock tables).

Table S1: Trial characteristics (mock table)

		Stratified by area of care[2]				
Variable	**RCTs assessing feasibility** (N = #)	**Critical care** (N = #)	**Emergency including prehospital care** (N = #)	**Anaesthesia and perioperative care including surgical interventions** (N = #)	**Non-critical non-emergent interventions on the ward** (N= #)	**Other areas** (N = #)
**Year of publication**[1]						
- 2025	n (#.#%)	n (#.#%)	n (#.#%)	n (#.#%)	n (#.#%)	n (#.#%)
**Number of sites**	## (## to ##) [## to ##]	## (## to ##) [## to ##]	## (## to ##) [## to ##]	## (## to ##) [## to ##]	## (## to ##) [## to ##]	## (## to ##) [## to ##]
**Number of participants**	## (## to ##) [## to ##]	## (## to ##) [## to ##]	## (## to ##) [## to ##]	## (## to ##) [## to ##]	## (## to ##) [## to ##]	## (## to ##) [## to ##]
**Area of care**[2]						
- Critical care	n (#.#%)	n (100.0%)	0 (0.0%)	0 (0.0%)	0 (0.0%)	0 (0.0%)
- Emergency including prehospital care	n (#.#%)	0 (0.0%)	n (100.0%)	0 (0.0%)	0 (0.0%)	0 (0.0%)
- Anaesthesia and perioperative care including surgical interventions	n (#.#%)	0 (0.0%)	0 (0.0%)	n (100.0%)	0 (0.0%)	0 (0.0%)
- Non-critical non-emergent interventions on the ward	n (#.#%)	0 (0.0%)	0 (0.0%)	0 (0.0%)	n (100.0%)	0 (0.0%)
- Other areas	n (#.#%)	0 (0.0%)	0 (0.0%)	0 (0.0%)	0 (0.0%)	n (100.0%)
**Participant age group**						
- Adult	n (#.#%)	n (#.#%)	n (#.#%)	n (#.#%)	n (#.#%)	n (#.#%)
- Children	n (#.#%)	n (#.#%)	n (#.#%)	n (#.#%)	n (#.#%)	n (#.#%)
- Mixed	n (#.#%)	n (#.#%)	n (#.#%)	n (#.#%)	n (#.#%)	n (#.#%)
**Number of intervention groups**[3]						
- 2	n (#.#%)	n (#.#%)	n (#.#%)	n (#.#%)	n (#.#%)	n (#.#%)
- More than 2	n (#.#%)	n (#.#%)	n (#.#%)	n (#.#%)	n (#.#%)	n (#.#%)
**Intervention type**						
- Drug	n (#.#%)	n (#.#%)	n (#.#%)	n (#.#%)	n (#.#%)	n (#.#%)
- Device	n (#.#%)	n (#.#%)	n (#.#%)	n (#.#%)	n (#.#%)	n (#.#%)
- Management	n (#.#%)	n (#.#%)	n (#.#%)	n (#.#%)	n (#.#%)	n (#.#%)
**Comparator type**						
- Active comparator	n (#.#%)	n (#.#%)	n (#.#%)	n (#.#%)	n (#.#%)	n (#.#%)
- No treatment	n (#.#%)	n (#.#%)	n (#.#%)	n (#.#%)	n (#.#%)	n (#.#%)

- Placebo or sham intervention	n (#.#%)	n (#.#%)	n (#.#%)	n (#.#%)	n (#.#%)	n (#.#%)
**Blinding**						
- Participants	n (#.#%)	n (#.#%)	n (#.#%)	n (#.#%)	n (#.#%)	n (#.#%)
- Care providers	n (#.#%)	n (#.#%)	n (#.#%)	n (#.#%)	n (#.#%)	n (#.#%)
- Outcome assessors	n (#.#%)	n (#.#%)	n (#.#%)	n (#.#%)	n (#.#%)	n (#.#%)
**Full protocol published**	n (#.#%)	n (#.#%)	n (#.#%)	n (#.#%)	n (#.#%)	n (#.#%)
**Pilot results published**	n (#.#%)	n (#.#%)	n (#.#%)	n (#.#%)	n (#.#%)	n (#.#%)
**Feasibility assessed**	n (100.0%)	n (100.0%)	n (100.0%)	n (100.0%)	n (100.0%)	n (100.0%)

Trial characteristics – total for RCTs assessing feasibility and stratified by area of care.
Categorical and binary variables are presented as counts (percentages). Numerical variables are presented as medians (interquartile range) [full ranges].
Trial characteristics to be extracted are DOIs*, trial name*, year of publication, country of origin*, number of sites, start of participant recruitment*, end of participant recruitment*, number of participants randomised, area of care, participant age group, number of intervention groups, intervention type, comparator type, blinding, publication of full protocol, publication of pilot trial results, and whether feasibility was assessed (i.e., assessment of trial design/conduct/process variables and not (only) of clinical outcomes registered at the individual participant level).
Characteristics marked with an asterix (*) are not shown in the table and will be reported in an appendix to the result manuscript. Definitions of all variables and categorisations are provided in the Supporting Information (Appendix B).
[1] Year of publication is registered for each RCT and will be reported in an appendix to the result manuscript. Because most RCTs are expected to be published in 2025, we will present it as a categorical variable with two levels (publication in 2025 or not). If a substantial number of RCTs are not published in 2025, we may choose to present it differently.
[2] The categorisation of care area is subject to change during data extraction if deemed relevant, i.e., new categories may be defined if a lot of trials are within areas of care not meaningfully covered by the present categories. Area of care for each included RCT will be presented in detail in a separate table in an appendix to the result manuscript.
[3] Number of intervention groups is presented as a categorical variable with two levels (two or more than two). The raw number of intervention groups in each RCT will be registered and reported in an appendix to the result manuscript, and if deemed relevant we will report counts and percentages for more than two levels.
Abbreviations: RCT: randomised clinical trial; DOI: digital object identifiers.

Table S2: Trial characteristics (mock table)

Variable	**RCTs assessing feasibility** (N = #)	*Stratified by comparator type* **Active comparator** (N = #)	**No treatment** (N= #)	**Placebo or sham intervention** (N = #)
**Year of publication**[1]				
- 2025	n (#.#%)	n (#.#%)	n (#.#%)	n (#.#%)
**Number of sites**	## (## to ##) [## to ##]	## (## to ##) [## to ##]	## (## to ##) [## to ##]	## (## to ##) [## to ##]
**Number of participants**	## (## to ##) [## to ##]	## (## to ##) [## to ##]	## (## to ##) [## to ##]	## (## to ##) [## to ##]
**Area of care**[2]				
- Critical care	n (#.#%)	n (#.#%)	n (#.#%)	n (#.#%)
- Emergency including prehospital care	n (#.#%)	n (#.#%)	n (#.#%)	n (#.#%)
- Anaesthesia and perioperative care including surgical interventions	n (#.#%)	n (#.#%)	n (#.#%)	n (#.#%)
- Non-critical non-emergent interventions on the ward	n (#.#%)	n (#.#%)	n (#.#%)	n (#.#%)
- Other areas	n (#.#%)	n (#.#%)	n (#.#%)	n (#.#%)
**Participant age group**				
- Adult	n (#.#%)	n (#.#%)	n (#.#%)	n (#.#%)
- Children	n (#.#%)	n (#.#%)	n (#.#%)	n (#.#%)
- Mixed	n (#.#%)	n (#.#%)	n (#.#%)	n (#.#%)
**Number of intervention groups**[3]				
- 2	n (#.#%)	n (#.#%)	n (#.#%)	n (#.#%)
- More than 2	n (#.#%)	n (#.#%)	n (#.#%)	n (#.#%)
**Intervention type**				
- Drug	n (#.#%)	n (#.#%)	n (#.#%)	n (#.#%)
- Device	n (#.#%)	n (#.#%)	n (#.#%)	n (#.#%)
- Management	n (#.#%)	n (#.#%)	n (#.#%)	n (#.#%)
**Comparator type**				
- Active comparator	n (#.#%)	n (100.0%)	0 (0.0%)	0 (0.0%)
- No treatment	n (#.#%)	0 (0.0%)	n (100.0%)	0 (0.0%)
- Placebo or sham intervention	n (#.#%)	0 (0.0%)	0 (0.0%)	n (100.0%)
**Blinding**				
- Participants	n (#.#%)	n (#.#%)	n (#.#%)	n (#.#%)
- Care providers	n (#.#%)	n (#.#%)	n (#.#%)	n (#.#%)
- Outcome assessors	n (#.#%)	n (#.#%)	n (#.#%)	n (#.#%)
**Full protocol published**	n (#.#%)	n (#.#%)	n (#.#%)	n (#.#%)
**Pilot results published**	n (#.#%)	n (#.#%)	n (#.#%)	n (#.#%)
**Feasibility assessed**	n (100.0%)	n (100.0%)	n (100.0%)	n (100.0%)

Trial characteristics – total for RCTs assessing feasibility and stratified by comparator type.
Categorical and binary variables are presented as counts (percentages). Numerical variables are presented as medians (interquartile range) [full ranges].

Trial characteristics to be extracted are DOIs*, trial name*, year of publication, country of origin*, number of sites, start of participant recruitment*, end of participant recruitment*, number of participants randomised, area of care, participant age group, number of intervention groups, intervention type, comparator type, blinding, publication of full protocol, publication of pilot trial results, and whether feasibility was assessed (i.e., assessment of trial design/conduct/process variables and not (only) of clinical outcomes registered at the individual participant level). Characteristics marked with an asterix (*) are not shown in the table and will be reported in an appendix to the result manuscript. Definitions of all variables and categorisations are provided in the Supporting Information (Appendix B).

[1] Year of publication is registered for each RCT and will be reported in an appendix to the result manuscript. Because most RCTs are expected to be published in 2025, we will present it as a categorical variable with two levels (publication in 2025 or not). If a substantial number of RCTs are not published in 2025, we may choose to present it differently.

[2] The categorisation of care area is subject to change during data extraction if deemed relevant, i.e., new categories may be defined if a lot of trials are within areas of care not meaningfully covered by the present categories. Area of care for each included RCT will be presented in detail in a separate table in an appendix to the result manuscript.

[3] Number of intervention groups is presented as a categorical variable with two levels (two or more than two). The raw number of intervention groups in each RCT will be registered and reported in an appendix to the result manuscript, and if deemed relevant we will report counts and percentages for more than two levels.

Abbreviations: RCT: randomised clinical trial; DOI: digital object identifiers.

Table S3: Feasibility assessment methodology (mock table)

		Stratified by area of care[2]				
Variable	**RCTs assessing feasibility** (N = #)	**Critical care** (N = #)	**Emergency including prehospital care** (N = #)	**Anaesthesia and perioperative care including surgical interventions** (N = #)	**Non-critical non-emergent interventions on the ward** (N = #)	**Other areas** (N = #)
**Stand-alone pilot**	n (#.#%)	n (#.#%)	n (#.#%)	n (#.#%)	n (#.#%)	n (#.#%)
**Feasibility area(s) assessed**[1]						
- Recruitment	n (#.#%)	n (#.#%)	n (#.#%)	n (#.#%)	n (#.#%)	n (#.#%)
- Randomisation procedure	n (#.#%)	n (#.#%)	n (#.#%)	n (#.#%)	n (#.#%)	n (#.#%)
- Consent	n (#.#%)	n (#.#%)	n (#.#%)	n (#.#%)	n (#.#%)	n (#.#%)
- Blinding procedures	n (#.#%)	n (#.#%)	n (#.#%)	n (#.#%)	n (#.#%)	n (#.#%)
- Protocol adherence	n (#.#%)	n (#.#%)	n (#.#%)	n (#.#%)	n (#.#%)	n (#.#%)
- Between-group separation	n (#.#%)	n (#.#%)	n (#.#%)	n (#.#%)	n (#.#%)	n (#.#%)
- Retention and attrition	n (#.#%)	n (#.#%)	n (#.#%)	n (#.#%)	n (#.#%)	n (#.#%)
- Other	n (#.#%)	n (#.#%)	n (#.#%)	n (#.#%)	n (#.#%)	n (#.#%)
**Outcomes pre-defined**	n (#.#%)	n (#.#%)	n (#.#%)	n (#.#%)	n (#.#%)	n (#.#%)
**Progression criteria pre-specified**	n (#.#%)	n (#.#%)	n (#.#%)	n (#.#%)	n (#.#%)	n (#.#%)
**Sample size adequately justified**	n (#.#%)	n (#.#%)	n (#.#%)	n (#.#%)	n (#.#%)	n (#.#%)
**Preparing for larger definitive RCT**	n (#.#%)	n (#.#%)	n (#.#%)	n (#.#%)	n (#.#%)	n (#.#%)
**Clinical outcomes assessed**						
- All intervention groups combined	n (#.#%)*	n (#.#%)*	n (#.#%)*	n (#.#%)*	n (#.#%)*	n (#.#%)*
- Separately by intervention group	n (#.#%)*	n (#.#%)*	n (#.#%)*	n (#.#%)*	n (#.#%)*	n (#.#%)*
- No	n (#.#%)*	n (#.#%)*	n (#.#%)*	n (#.#%)*	n (#.#%)*	n (#.#%)*
**Trial deemed feasible**						
- Yes	n (#.#%)*	n (#.#%)*	n (#.#%)*	n (#.#%)*	n (#.#%)*	n (#.#%)*
- With modifications	n (#.#%)*	n (#.#%)*	n (#.#%)*	n (#.#%)*	n (#.#%)*	n (#.#%)*
- No	n (#.#%)*	n (#.#%)*	n (#.#%)*	n (#.#%)*	n (#.#%)*	n (#.#%)*

Feasibility assessment methodology – total for RCTs assessing feasibility and stratified by area of care.
Categorical and binary variables are presented as counts (percentages).
*Percentages to be reported are the percentages calculated using the number of *trials with published pilot trial results* as the denominator.
[1] The categorisation of feasibility areas is subject to change during data extraction if deemed relevant, i.e., new categories may be defined if a lot of trials assess areas not meaningfully covered by the present categories. Area of feasibility assessment for each included RCT will be presented in separate table in an appendix to the result manuscript.
[2] The categorisation of care area is subject to change during data extraction if deemed relevant, i.e., new categories may be defined if a lot of trials are within areas of care not meaningfully covered by the present categories. Area of care for each included RCT will be presented in detail in a separate table in an appendix to the result manuscript.
Abbreviations: RCT: randomised clinical trial.

Table S4: Feasibility assessment methodology (mock table)

Variable	RCTs assessing feasibility (N = #)	*Stratifed by comparator type* Active comparator (N = #)	No treatment (N = #)	Placebo or sham intervention (N = #)
**Stand-alone pilot**	n (#.#%)	n (#.#%)	n (#.#%)	n (#.#%)
**Feasibility area(s) assessed**[1]				
- Recruitment	n (#.#%)	n (#.#%)	n (#.#%)	n (#.#%)
- Randomisation procedure	n (#.#%)	n (#.#%)	n (#.#%)	n (#.#%)
- Consent	n (#.#%)	n (#.#%)	n (#.#%)	n (#.#%)
- Blinding procedures	n (#.#%)	n (#.#%)	n (#.#%)	n (#.#%)
- Protocol adherence	n (#.#%)	n (#.#%)	n (#.#%)	n (#.#%)
- Between-group separation	n (#.#%)	n (#.#%)	n (#.#%)	n (#.#%)
- Retention and attrition	n (#.#%)	n (#.#%)	n (#.#%)	n (#.#%)
- Other	n (#.#%)	n (#.#%)	n (#.#%)	n (#.#%)
**Outcomes pre-defined**	n (#.#%)	n (#.#%)	n (#.#%)	n (#.#%)
**Progression criteria pre-specified**	n (#.#%)	n (#.#%)	n (#.#%)	n (#.#%)
**Sample size adequately justified**	n (#.#%)	n (#.#%)	n (#.#%)	n (#.#%)
**Preparing for larger definitive RCT**	n (#.#%)	n (#.#%)	n (#.#%)	n (#.#%)
**Clinical outcomes assessed**				
- All intervention groups combined	n (#.#%)*	n (#.#%)*	n (#.#%)*	n (#.#%)*
- Separately by intervention group	n (#.#%)*	n (#.#%)*	n (#.#%)*	n (#.#%)*
- No	n (#.#%)*	n (#.#%)*	n (#.#%)*	n (#.#%)*
**Trial deemed feasible**				
- Yes	n (#.#%)*	n (#.#%)*	n (#.#%)*	n (#.#%)*
- With modifications	n (#.#%)*	n (#.#%)*	n (#.#%)*	n (#.#%)*
- No	n (#.#%)*	n (#.#%)*	n (#.#%)*	n (#.#%)*

Feasibility assessment methodology – total for RCTs assessing feasibility and stratified by comparator type.

Categorical and binary variables are presented as counts (percentages).

*Percentages to be reported are the percentages calculated using the number of *trials with published pilot trial results* as the denominator.

[1] The categorisation of feasibility areas is subject to change during data extraction if deemed relevant, i.e., new categories may be defined if a lot of trials assess areas not meaningfully covered by the present categories. Area of feasibility assessment for each included RCT will be presented in separate table in an appendix to the result manuscript.

Abbreviations: RCT: randomised clinical trial.

References

1. Cochrane Handbook for Systematic Reviews of Interventions (current version) | Cochrane [Internet]. [cited 2025 Oct 26]. Available from: https://www.cochrane.org/authors/handbooks-and-manuals/handbook/current

2. Moher D, Shamseer L, Clarke M, Ghersi D, Liberati A, Petticrew M, Shekelle P, Stewart LA, PRISMA-P Group. Preferred reporting items for systematic review and meta-analysis protocols (PRISMA-P) 2015 statement. Syst Rev 2015; 4: 1.

3. Murad MH, Wang Z. Guidelines for reporting meta-epidemiological methodology research. Evid Based Med 2017; 22: 139–42.
